# Artificial Intelligence Goes Physical

**DOI:** 10.1002/smsc.202000065

**Published:** 2021-02-15

**Authors:** Zhaokun Jing, Yuchao Yang

**Affiliations:** ^1^ Key Laboratory of Microelectronic Devices and Circuits (MOE) Department of Micro/nanoelectronics Peking University Beijing 100871 China; ^2^ Center for Brain Inspired Chips Institute for Artificial Intelligence Peking University Beijing 100871 China

**Keywords:** artificial intelligence, nonlinearity, noise, reservoir computing

## Abstract

Exploiting the intrinsic nonlinearity in physical reservoirs, e.g., dopant‐atom networks, provides a new approach toward highly efficient computing such as feature projection and classification. In a recent study by Chen et al., the computational capability of dopant‐atom network was investigated and found to diminish as the signal‐to‐noise ratio (SNR) increased, indicating the existence of an optimal bias condition. Although high SNR is often pursued in signal processing, it shows that embracing noise in non‐conventional computing systems may lead to a leap in computing capacity. This work showcased that material or device physics in different domains offer valuable substrates for complex computing functions and high energy efficiency.

## Graphical abstract

Conventional computing platforms with nonlinear functionality usually require complicated circuits and control logic, thus consuming extensive chip area and energy. Exploration of the intrinsic nonlinearity in physical reservoirs provides a new approach toward building highly area‐ and energy‐efficient computing hardware with complex functionality. In particular, embracing noise in nonconventional computing systems may lead to a leap in computing capacity.

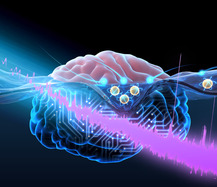

Conventional computing platforms with nonlinear functionality usually require complicated circuits and control logic, thus consuming extensive chip area and energy for large‐scale computational tasks. On the contrary, exploring the intrinsic nonlinearity in physical systems provides a new approach toward building highly area‐ and energy‐efficient computing hardware with complex functionality. Recent years have witnessed remarkable progress in physical reservoir computing,^[^ ^1^ ^]^ which implements nonlinear feature projection in the reservoir using a variety of physical phenomena in the real world. Recently, a disordered dopant‐atom network in silicon^[^ ^2^ ^]^ is proposed to solve nonlinear classification problems with over 100 TOPS W^−1^ projected energy efficiency and 300 nm × 300 nm area. This dopant‐atom network exploits the nonlinear electron hopping characteristics as a reservoir by configuring input and control voltages and therefore also falls into the category of physical reservoir computing systems. However, the system working under electron hopping regime is naturally coupled with noises, resulting in output current fluctuations. Therefore, the influence of noise over the computing performance of the system, e.g., signal‐to‐noise ratio (SNR) and nonlinearity, is an important concern.

These questions have attracted wide interests,^[^ ^1^, ^2^, ^3^ ^]^ and in a recent study, a detailed investigation was conducted to address them.^[^ ^3^ ^]^ Chen et al. experimentally confirmed that the computational capability of the dopant‐atom network, i.e., nonlinearity, diminishes with rising SNR, and the varied SNR is due to different scaling effects of the response current and 1/*f* noise intensity with bias voltage. Further analysis indicates the optimal bias condition of the dopant‐atom network, which can be potentially generalized to other complex nonlinear systems.

**Figure 1:**
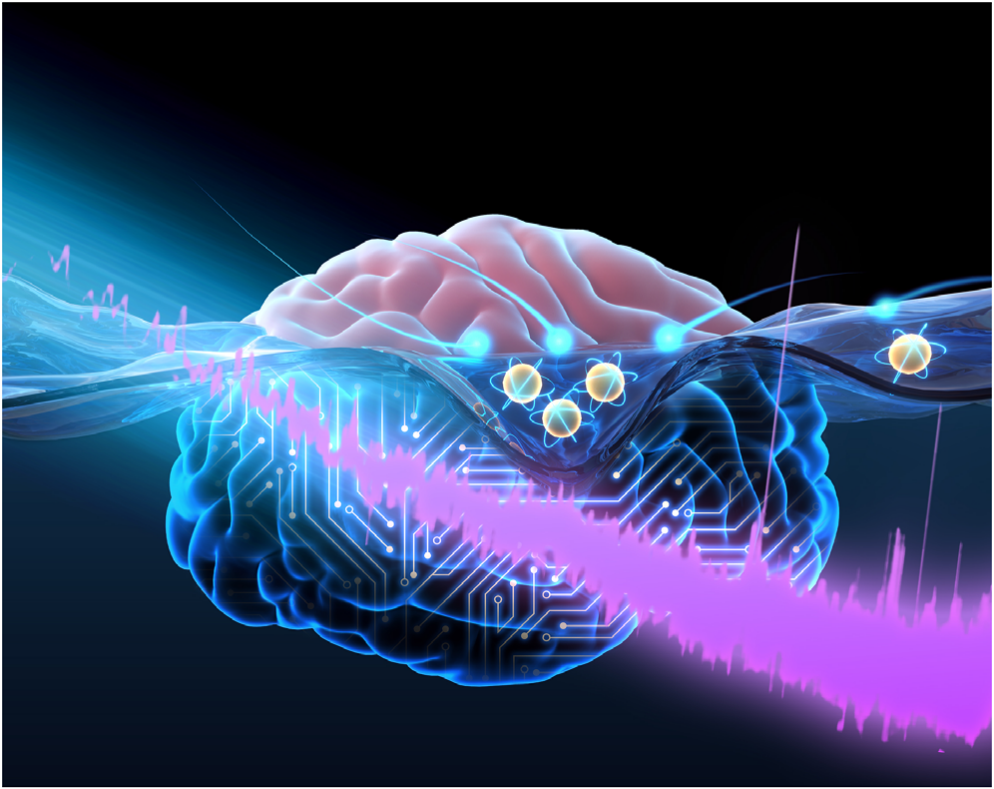
Embracing noise in nonconventional computing systems may lead to a leap in computing capacity.

In general, noise is considered undesirable in computing systems, and high SNR is always pursued in signal processing.^[^ ^4^ ^]^ The interesting point of the work by Chen et al. is that it shows powerful computing functionality is concomitant with noises in physical systems. Embracing noise in nonconventional computing systems may lead to a leap in computing capacity (**Figure** **1**).

Recently, computing systems based on emerging nonvolatile devices such as resistive random access memories (RRAMs)^[^ ^5^ ^]^ and phase change memories^[^ ^6^ ^]^ have shown great potential in enabling a new generation of computing architectures, such as compute‐in‐memory architecture,^[^ ^7^ ^]^ but the intrinsic noise in such devices^[^ ^8^, ^9^ ^]^ may also deteriorate the system performance and require careful engineering.^[^ ^10^ ^]^

On the contrary, some attempts have been devoted to utilize noise characteristics of the device instead as computing resources. For instance, the intrinsic random noise of RRAM devices can be utilized as the input of a generative adversarial network,^[^ ^11^ ^]^ which is helpful for mitigating mode collapse problems when training the network. Similarly, the intrinsic analog noise in RRAM crossbars can be leveraged for the implementation of a simulated annealing algorithm.^[^ ^12^ ^]^

It is interesting to find that 1/*f* noise is also commonly found in brain activities^[^ ^13^ ^]^ and is believed to be the consequence of complex neural dynamics.^[^ ^13^ ^,^ ^14^ ^]^ Although the correlation between 1/*f* noise and cognitive functionality of the brain remains elusive, the latest findings provide evidence that existence of noise in complex computing systems is possibly an indication of highly nonlinear computational functionality. The tradeoff between SNR and system functionality may exist in a variety of physical systems, and there can be vast possibilities to explore the “material intelligence.”

It should be noted that a prevailing approach to the construction of intelligent systems at present is to exploit the nonvolatile (or even analog) states of emerging memory devices so as to efficiently perform the multiply‐accumulate (MAC) functions based on Ohm's law and Kirchhoff's current law. Despite the encouraging progresses, this method only accelerates linear operations, and the nonlinearity required is provided externally in circuit or software. Such architecture can be seen as a “top‐down” design of deep learning or machine learning algorithms, and therefore faces the same limitations of existing algorithms. In this regard, physical systems with inherent computational functionalities may offer building blocks for the construction of a different type of intelligent systems. Apart from the aforementioned Ohm's law and Kirchhoff's current law, material or device physics in other domains may be exploited as the substrate of physical computing, such as stochasticity, physical evolution, or temporal dynamics. Combining these nonlinear physical units with a “bottom‐up” design methodology may stand a chance in achieving more complex computing functions and extremely high energy efficiency.

## Conflict of Interest

The authors declare no conflict of interest.

## Data Availability Statement

Data sharing is not applicable to this article as no new data were created or analyzed in this study.
